# Acknowledgement to Reviewers of *Journal of Intelligence* in 2017

**DOI:** 10.3390/jintelligence6010003

**Published:** 2018-01-19

**Authors:** J. Intell. Editorial Office

**Affiliations:** MDPI AG, St. Alban-Anlage 66, 4052 Basel, Switzerland; jintelligence@mdpi.com

Peer review is an essential part in the publication process, ensuring that *Journal of Intelligence* maintains high quality standards for its published papers. In 2017, a total of 31 papers were published in the journal. Thanks to the cooperation of our reviewers, the median time to first decision was 39 days and the median time to publication was 98 days. The editors would like to express their sincere gratitude to the following reviewers for their time and dedication in 2017:
Abele, StephanHalpin, Peter F.Raitasalo, KirsimarjaAndenoro, TonyHarrison, VirginiaRanger, JochenAnderson, MikeHass, Richard W.Redifer, JenniBaudson, Tanja GabrieleHernandez-Orallo, JoseReisenzein, RainerBayen, Ute J.Hill, DavidRey-Mermet, AlodieBeckmann, Jens F.Horn, SebastianRinaldi, LucaBrewer, GeneHülür, GizemRindermann, HeinerBriley, Daniel A.Jaeggi, SuzanneRoos, MicahBuchner, AxelJohnson, WendyRosen, YigalCastejon Costa, Juan LuisKan, Kees-JanRothstein, MitchCeci, StephenKandler, ChristianRuch, WillibaldCheatham, CarolKell, Harrison J.Schlegel, KatjaCheung, AmandaKievit, RogierSchmidt, FrankColom, RobertoKovacs, KristofSchmiedek, FlorianConway, AndrewKretzschmar, AndréSchmitz, FlorianCostello, ShaneKröner, StephanSchneider, W. JoelCoyle, TomKyllonen, Patrick C.Schroeders, UlrichDauvier, BrunoLegree, Peter J.Sijtsma, KlaasDe Boeck, PaulLemaire, PatrickSonnleitner, PhilippDe Ribaupierre, AnikLencz, ToddSt Clair-Thompson, HelenDoebler, PhilippLi, WendongStadler, MatthiasElliott, EmilyMacDonald, StuartStawski, RobertFinkel, DeborahMackey, AllysonSternberg, RobertFiore, Stephen M.Maier, SimonSternberg, Robert J.Fischer, AndreasMarcoulides, George A.Stevenson, ClaireFloyd, RandyMarzoli, DanieleSwanson, H. LeeFontaine, JohnnyMcGill, Ryan J.Trapero, IsabelFrank, Brian M.Meyers, RaymondTremblay, PaulGade, MiriamMok, DianaTSE, Chi-ShingGanzach, Yo'avMolenaar, DylanVan Der Maas, Han L.J.Ghisletta, PaoloMurphy, KevinVerhaeghen, PaulGignac, GillesNęcka, EdwardVoss, AndreasGiofre, DavidNeubauer, AndreasWatkins, Marley W.Goldhammer, FrankNiklas, FrankWhitaker, KirstieGrant, MichaelOkbay, AysuWilhelm, OliverGreiff, SamuelPerret, PatrickWoodley, MichaelGrigorenko, Elena L.Pillow, David R.Zehner, FabianGüss, DominikPlomin, RobertZeidner, MosheHajovsky, DanielPrice, PatriciaZiegler, Matthias

